# Brain Functional Connectivity Is Modified by a Hypocaloric Mediterranean Diet and Physical Activity in Obese Women

**DOI:** 10.3390/nu9070685

**Published:** 2017-07-01

**Authors:** Natalia García-Casares, María R. Bernal-López, Nuria Roé-Vellvé, Mario Gutiérrez-Bedmar, Jose C. Fernández-García, Juan A. García-Arnés, José R. Ramos-Rodriguez, Francisco Alfaro, Sonia Santamaria-Fernández, Trevor Steward, Susana Jiménez-Murcia, Isabel Garcia-Garcia, Pedro Valdivielso, Fernando Fernández-Aranda, Francisco J. Tinahones, Ricardo Gómez-Huelgas

**Affiliations:** 1 Department of Medicine, Faculty of Medicine, University of Málaga, Málaga 29010, Spain; valdivielso@uma.es (P.V.); ricardogomezhuelgas@hotmail.com (R.G.-H.); 2 Centro de Investigaciones Médico-Sanitarias (C.I.M.E.S), University of Málaga, Málaga 29010, Spain; nroe@fguma.es (N.R.-V.); ramosmenoyo@gmail.com (J.R.R.-R.); alfarorubio@hotmail.com (F.A.); 3 Department of Internal Medicine, University Regional Hospital, Málaga; Instituto de Investigación Biomédica de Málaga (IBIMA), Málaga 29010, Spain; robelopajiju@yahoo.es (M.R.B.-L.); eclyris@hotmail.com (S.S.-F.); 4 CIBER Fisiopatología de la Obesidad y Nutrición (CIBERObn), Instituto Salud Carlos III (ISCIII), Madrid 28029, Spain; josecarlosfdezgarcia@hotmail.com (J.C.F.-G.); tsteward@bellvitgehospital.cat (T.S.); sjimenez@bellvitgehospital.cat (S.J.-M.); ffernandez@bellvitgehospital.cat (F.F.-A.); 5 Department of Public Health and Psychiatry, University of Málaga, Málaga 29010, Spain; bedmar@uma.es; 6 Department of Endocrinology, Virgen de la Victoria University Hospital, Málaga; Instituto de Investigación Biomédica de Málaga (IBIMA), Málaga 29010, Spain; 7 Department of Endocrinology, Regional University Hospital, Málaga 29010, Spain; arnes@uma.es; 8 Department of Psychiatry, Bellvitge University Hospital-IDIBELL, Hospitalet de Llobregat 08908, Spain; 9 Department of Clinical Sciences, School of Medicine, University of Barcelona, l’Hospitalet de Llobregat 08907, Spain; 10 Department of Neurology and Neurosurgery, Montreal Neurological Institute, McGill University, Montreal, QC 3801, Canada; isabel.garciagarcia@mcgill.ca; 11 Department of Internal Medicine, Virgen de la Victoria University Hospital, Málaga; Instituto de Investigación Biomédica de Málaga (IBIMA), Málaga 29010, Spain

**Keywords:** Mediterranean diet, physical activity, connectivity, obesity, inferior parietal lobe, temporal lobe, insula, prefrontal cortex, posterior cingulate, somatosensory cortex, functional MRI, resting state

## Abstract

Functional magnetic resonance imaging (fMRI) in the resting state has shown altered brain connectivity networks in obese individuals. However, the impact of a Mediterranean diet on cerebral connectivity in obese patients when losing weight has not been previously explored. The aim of this study was to examine the connectivity between brain structures before and six months after following a hypocaloric Mediterranean diet and physical activity program in a group of sixteen obese women aged 46.31 ± 4.07 years. Before and after the intervention program, the body mass index (BMI) (kg/m^2^) was 38.15 ± 4.7 vs. 34.18 ± 4.5 (*p* < 0.02), and body weight (kg) was 98.5 ± 13.1 vs. 88.28 ± 12.2 (*p* < 0.03). All subjects underwent a pre- and post-intervention fMRI under fasting conditions. Functional connectivity was assessed using seed-based correlations. After the intervention, we found decreased connectivity between the left inferior parietal cortex and the right temporal cortex (*p* < 0.001), left posterior cingulate (*p* < 0.001), and right posterior cingulate (*p* < 0.03); decreased connectivity between the left superior frontal gyrus and the right temporal cortex (*p* < 0.01); decreased connectivity between the prefrontal cortex and the somatosensory cortex (*p* < 0.025); and decreased connectivity between the left and right posterior cingulate (*p* < 0.04). Results were considered significant at a voxel-wise threshold of *p* ≤ 0.05, and a cluster-level family-wise error correction for multiple comparisons of *p* ≤ 0.05. In conclusion, functional connectivity between brain structures involved in the pathophysiology of obesity (the inferior parietal lobe, posterior cingulate, temporo-insular cortex, prefrontal cortex) may be modified by a weight loss program including a Mediterranean diet and physical exercise.

## 1. Introduction

Studies of the pathophysiology of obesity using advanced neuroimaging techniques such as structural magnetic resonance imaging (MRI), functional MRI (fMRI) (at rest or using tasks), and positron emission tomography have found altered brain systems related to reward, motor, cognition, control, and attention [1,2]. Other studies have demonstrated that a Mediterranean diet has cognitive benefits [3], suggesting that it may have a potential role in preventing overweight/obesity [4,5], though most of these were clinical studies with no neuroimaging analysis. Neuroimaging markers are sensitive measurements of structural and functional changes in the aging brain. We can use these markers to examine associations between diet- and age-related brain changes that might not be detectable using clinical assessment. A few studies have examined the relationship between a Mediterranean diet and structural neuroimaging markers of gray matter volume such as brain atrophy and cortical thickness [6,7,8,9,10,11,12]. Luciano et al. found that lower adherence to a Mediterranean diet was associated with total brain atrophy over a three-year interval [6], and Gu et al. demonstrated that Mediterranean diet adherence was associated with less brain atrophy, with larger brain volumes of the cingulate cortex, parietal lobe, temporal lobe, and hippocampus, and larger cortical thickness of the superior-frontal region [7]. Other studies found similar results, showing evidence that the Mediterranean diet predominantly protects the brain structure in certain cognitively-vulnerable regions (e.g., the frontotemporal lobes) [9,10]. In addition, the relationship between the Mediterranean diet and white matter structures has also been explored. Pelletier et al. found that Mediterranean diet adherence was associated with a general pattern of preserved white matter microstructure in multiple regions [11]. Other studies have also shown the benefits of diet and physical activity on brain structure in successful dieters. Honea et al. found that weight loss percentage correlated positively with baseline gray matter volume in the right parahippocampal and orbitofrontal gyri in obese dieters, concluding that weight loss is related to volumetric changes in brain areas linked to interoception and food motivation [12]. On the other hand, some studies have examined obesity and cerebral connectivity through functional magnetic resonance with a resting state modality, but very few included a dietary intervention and/or physical exercise. Furthermore, the impact of a Mediterranean diet on cerebral connectivity in obese patients when losing weight has not been previously explored. Thus, this study includes resting state and an intervention with a Mediterranean diet. Brain functional connectivity studies in the resting state in obesity are emerging, and have focused attention on brain networks such as the default mode network (DMN), the salience network (SN), and the somatosensory network (SMN) [13,14,15,16,17] as being networks susceptible to alteration.

The DMN is a functionally-connected network of brain regions that includes the posterior cingulate cortex, cuneus/precuneus, medial prefrontal cortex, medial temporal lobe, and inferior parietal cortices [18]. Connectivity in the DMN is thought to reflect a baseline state of brain function, in which subjects are not focused on the external environment but rather on their internal mental state, which may include various forms of spontaneous cognition, self-reflective thought, or attention to internal stimuli [18]. Previous resting-state studies have demonstrated potential differences in the DMN function between obese and lean subjects, with an increased DMN connectivity in obese individuals [14]. In addition, exercise has been found to normalize DMN function in overweight/obese individuals [16].

The SN comprises paralimbic structures such as the dorsal anterior cingulate cortex (dACC) and the orbital and insular cortices [19], and is involved in emotional arousal, reward sensitivity, and decision-making [19]. Functional connectivity in this network has also been found to be altered in obesity [20]. Under resting-state screening, functional connectivity changes in the SN have been reported in obese as compared with lean individuals [15,21]. Increased connectivity in SN structures such as the nucleus accumbens and anterior cingulate has also been reported in obese women [22]. Another study showed increased functional connectivity within the putamen nucleus in the SN in obese subjects [22]. Finally, in response to visual food cues, chronic exercise has been associated with significantly reduced activation in the insula [23].

The SMN—including the supplementary motor area, sensorimotor cortex, and secondary somatosensory cortex [24]—has also been found to be altered in obesity [13,25]. In addition, associations have been found between these three networks (DMN, SN, and SMN) and fat mass and the obesity-associated gene Obesity Risk Allele during the resting-state [13].

The aim of this study was to examine the connectivity between structures included in the DMN, SN, and SMN before and six months after a hypocaloric Mediterranean diet and physical activity program in a group of obese women. Previous cross-sectional studies comparing obese with lean subjects have generally reported increased connectivity after studying these networks directly. Furthermore, in the present study we explored different structures included in these networks using seed-based analysis and examined the possible changes in connectivity between them before and after a weight loss program. We hypothesized that these obesity-related alterations in functional connectivity would be modified after the program, with obese subjects displaying decreased connectivity.

## 2. Materials and Methods

### 2.1. Participants

The study involved 19 obese women (defined by a body mass index (BMI, the weight in kilograms divided by the square of the height in meters) ≥30 kg/m^2^) who agreed to participate in a program comprising a hypocaloric weight-loss Mediterranean diet and physical activity. Participants were included in the study on their first visit if they met the following inclusion criteria: having no metabolic disorder related to obesity, women between the ages of 35 and 55, had not tried to lose weight within one year of our intervention, and whose lifestyle was sedentary. Exclusion criteria included a history of any neurological disorder, an estimated intelligence quotient <85 points (assessed with the vocabulary subtest of the Wechsler Adult Intelligence Scale 3rd edition (WAIS-III)), or the presence of any psychiatric disorder (including eating disorders, anxiety or depression assessed with the Hamilton anxiety and depression scale, abuse of smoking, alcohol, or other drugs abuse), use of medication that could affect brain function (e.g., antidepressants), a history of any metabolic disorder possibly related to obesity, and general contraindications for MRI. Participants were recruited from the Endocrinology Department at the Regional University Hospital of Malaga, and the MRIs were performed at the Centro de Investigaciones Médico Sanitarias (CIMES), University of Malaga. The study was performed in accordance with the principles of the revised Declaration of Helsinki. The protocol was approved by the Medical Ethics Committee of the Regional University Hospital of Malaga (approval code API5-10/13) on 29 October 2013, and all subjects provided written and signed informed consent before participation.

### 2.2. Intervention

The diet consisted of a hypocaloric Mediterranean diet [26,27] based on the use of olive oil as the main source of visible fat and a regular consumption of vegetables (≥2 servings/day), fruits (≥3 servings/day), legumes (≥3 servings/week), and fish (≥3 times per week), reducing the consumption of red meat (≤2 times per week), and eliminating (or reducing to ≤1 time/week) the consumption of dairy milk, sugary drinks, and sweets and chocolates. This hypocaloric Mediterranean diet restricted energy intake to about 600 kcal (or approximately 30% of the estimated energy requirement); 35–40% of the total daily calorie intake was from fat (8–10% saturated fatty acids), 40–45% carbohydrates (low glycemic index), and 20% protein based on the general patterns of the Mediterranean diet, calculating a calorie deficit following the Harris–Benedict equation, with the aim of achieving a body weight loss of ≥5%.

Participants were given healthy lifestyle guidelines by a nutritionist and were provided with a physical activity monitor. Regarding nutrition, participants made individual personalized visits with the nutritionist 30–60 min per week during the intervention, in which their body weight was measured and the response to weight loss was seen. In addition, they were given a validated semi-quantitative 137-item food frequency questionnaire [28,29] as well as a validated 14-item questionnaire [30] to measure the adherence to the Mediterranean diet both at the beginning and six months after starting the intervention. High adherence was considered to be 12–14 points, moderate adherence 8–11 points, low adherence 5–7 points, and very low adherence 5 points. Furthermore, nutritional group meetings were held monthly to increase the motivation of all participants. Concerning the physical activity program, a daily exercise practice was recommended. The minimum aim was walking an average of 150 min per week. This minimum level of defined activity was based on the cardiometabolic benefit demonstrated in other studies [31].

Participants were assessed and encouraged to gradually increase their level of physical activity to reach at least 45 min per day at the end of the six-month intervention period. In addition, participants were given a pedometer for their own use to encourage motivation. Once a month during the intervention, they went to the sports center with the physical activity monitor who checked whether the participants performed the physical activity.

Before, during, and after the whole program, weekly anthropometric measurements (weight, body mass index, and waist circumference) were taken with a Tanita BC-420MA (Tanita Corp., Tokyo, Japan), and height was measured by a stadiometer with the participants wearing no shoes. Blood analysis measurements, including glucose, total cholesterol, triglycerides, low-density lipoprotein (LDL) cholesterol, high-density lipoprotein (HDL) cholesterol, insulin, glycosylated hemoglobin (HbA1c), and insulin resistance index (HOMA-IR), were also recorded at baseline and after the program.

### 2.3. Study Design

All participants underwent functional magnetic resonance imaging (fMRI) scans under resting state conditions before and six months after following the Mediterranean diet and physical activity program. All the post-intervention fMRI scans were performed within 24–72 h after completing the intervention. They were asked to refrain from eating or drinking (except water) for five hours before the scan. All the MRI scans were performed at the same time in the afternoon. Functional connectivity of the regions of interest was assessed using a seed-based correlation approach. Three participants were not included in the analysis because they did not perform the second MRI (one did not complete the intervention and two did not want to be scanned again, although they completed the intervention). The rest of the participants (*n* = 16 women) responded to treatment and none gained weight.

### 2.4. Image Acquisition

The MRI scans were performed on a 3-T MRI scanner (Philips,Amsterdam,Netherlands, Medical Systems Netherlands, 3T Intera, Release 2.6, with an eight-channel platform) with a MASTER gradient system (nominal maximum gradient strength = 30 mT/m, maximum slew rate = 150 mT/m/ms), equipped with a six-channel Philips SENSE head coil. Head movements were minimized using head pads and a forehead strap. High-resolution T1 structural images of the whole brain were acquired using a matrix acquisition 312/320 r, field of view of 250 mm, Repetition Time (TR) = 9.9 ms, Echo Time (TE) = 4.5 ms, flip angle = 8°, Turbo field echo (TFE) factor = 179. Slice thickness was 0.8 mm and there was no gap between slices; voxels were isotropic: 0.8 mm × 0.8 mm × 0.8 mm.

Resting state fMRI (rs-fMRI) images were also acquired, with a Fast Field Echo (FFE)/ Echo planar imaging (EPI) sequence with a 220 mm field of view (FOV), TR = 2000 ms, TE= 35 ms, flip angle 82°, an acquisition matrix of 80/128 r, and an acquisition voxel size of 2.75 mm × 2.75 mm × 4.00 mm. Three hundred volumes were obtained with 32 slices each, with no gap between the slices. The length of time of the resting state acquisition was 10:00 min. Both structural and rs-fMRI studies were acquired before and after the intervention.

### 2.5. Image Processing

Image preprocessing was performed using statistical parametric mapping (SPM8, Wellcome Department of Imaging Neuroscience, London, England; www.fil.ion.ucl.ac.uk/spm) running on MATLAB 8.2.0.701 (R2013b). Each subject’s functional images were realigned to the mean position of all scans, and it was checked that the participants’ movements during acquisition did not exceed 1.7 mm translation or 2.3° rotation. High-resolution structural T1 images were anterior and posterior commissures (AC-PC) oriented, and then the functional scans were co-registered to the respective T1 images. These were used for normalization to the standard Montreal Neurological Institute (MNI) space. Smoothing of the functional scans was carried out with an 8-mm full width at half maximum (FWHM) kernel. The structural scans were also segmented in order to obtain grey matter (GM), white matter (WM), and cerebral-spinal fluid (CSF) maps.

The conn toolbox (version 1.4, McGovern Institute for Brain Research, Massachusetts Institute of Technology, Cambridge, USA, http://www.nitrc.org/projects/conn) was used for the seed-based connectivity analysis. A band-pass filter with cut-off frequencies of 0.008 and 0.09 Hz was applied to exclude drifts and high-frequency activations. In addition to movement parameters, white matter and CSF blood oxygen level-dependent (BOLD) time-series were also introduced as confounders in the denoising stage in order to exclude non-neural fluctuations from the analysis.

The areas selected as seeds were structures included in the well-established neural networks (DMN, SN, and SSN) including: left and right inferior parietal lobes, right and left superior temporal gyrus, left and right insula, right and left superior frontal cortex, medial prefrontal cortex, anterior cingulate gyrus, right and left posterior cingulate gyrus/precuneus, and right and left olfactory regions. Bilateral olfactory and posterior cingulum regions were taken from the IBASPM116 atlas included in the Wake Forest University Pickatlas toolbox [32]. The other areas were taken from the resting state reliability (rsREL) regions of interest (ROIs) provided with the conn toolbox. They are defined as 9-mm radius spheres, centered on the coordinates (seed definitions).

Following the usual pipeline for seed-based correlation analyses, a first-level individual analysis was initially carried out, followed by a second-level group analysis. Firstly, an ROI-to-ROI approach was used: the BOLD signal timeline at each seed region was checked for correlations with the BOLD signal timeline at each of the other preselected regions. Afterwards, a seed-to-voxel analysis was also carried out: the correlation between the BOLD timeline at the seed region and the BOLD timeline at each voxel of the image was calculated. As mentioned, these correlations were initially studied on an individual level, and then the second level of analysis was applied. Here, the parametric (*z*-score) values obtained for each individual were compared, both at the ROI-to-ROI and at the seed-to-voxel levels. In particular, a paired *t*-test allowed assessment of changes in BOLD signal correlation associated to the program for each seed.

For ROI-to-ROI analyses, two-sided family-wise error (FWE) correction was applied at the analysis level, with a significance threshold of *p* = 0.05. Seed-to-voxel comparisons were FWE-corrected and false discovery rate (FDR)-corrected at the cluster level, with the same threshold as before (*p* = 0.05).

### 2.6. Statistical Analysis

All the clinical and blood test parameters are summarized as descriptive statistics in Table 1. The results are presented as the mean ± standard deviation. For inter-group comparisons before and after the intensive weight loss program, paired-sample *t*-tests were used for continuous variables, and the Wilcoxon Rank Sum test for such variables when the assumptions for the *t*-test were violated. The α-level was set at *p* < 0.05 for these two-tailed statistical comparisons using SPSS statistical software, version 21.0 for Windows (SPSS Inc., Chicago, IL, USA).

## 3. Results

### 3.1. Demographic and Clinical Characteristics

The participants were 16 obese women aged 46.31 ± 4.07 years. Before and after the Mediterranean diet and physical activity program, the BMI (kg/m^2^) was 38.15 ± 4.7 vs. 34.18 ± 4.5 (*p* < 0.02), and the weight (kg) was 98.5 ± 13.1 vs. 88.28 ± 12.2 (*p* < 0.03). The score for adherence to the Mediterranean diet rose from 7.2 ± 2.3 points at baseline to 10.7 ± 2.0 points after the program. All the clinical, anthropometric, and blood analysis characteristics at baseline and after the weight loss program are summarized in Table 1, and changes in energy and nutrient intakes according to weight loss baseline and after the intervention are summarized in Table 2.

### 3.2. Neuroimaging Connectivity

#### 3.2.1. Functional Connectivity of the Left Inferior Parietal Lobe

**Figure 1:**
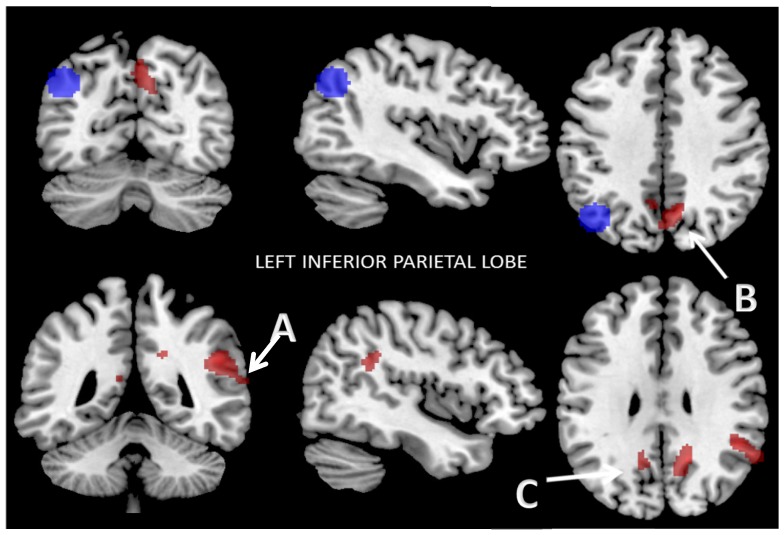
Reduced connectivity between left inferior parietal lobe (IPL) (blue) and the first cluster: (**A**) the right angular gyrus (AG); the right superior temporal gyrus (STG); the right supramarginal gyrus (SmG), and the insular cortex (IC) (*p* < 0.001); the second cluster (**B**) right dorsal posterior cingulate cortex (dpCC) and left and right somatosensory association cortices (SsAC) (*p* < 0.001); the third cluster: the left dpCC, the left SsAC, the left retrosplenial cingulate cortex (RsCC), and the left cingulate cortex (CC) (*p* < 0.03); (**C**) after the Mediterranean diet and physical activity program compared to baseline. Data are thresholded at a voxel-wise threshold of *p* ≤ 0.05 and a cluster-extent threshold of *p* ≤ 0.05, FWE-corrected and FDR-corrected.

In the ROI-to-ROI analysis, the left inferior parietal lobe (IPL) showed lower connectivity with the precuneus and the right inferior parietal lobe after the Mediterranean diet and weight loss program than before (two-sided FWE-correction at the analysis level, *p* < 0.05). In the seed-to-voxel examination, the IPL showed decreased functional connectivity with three clusters after the program. The first cluster included the right angular gyrus (AG), the right superior temporal gyrus (STG), the right supramarginal gyrus (SmG), and the insular cortex (IC) (*p* < 0.001); the second cluster included the right dorsal posterior cingulate cortex (dpCC) and the left and right somatosensory association cortices (SsAC) (*p* < 0.001); and the third cluster included the left dpCC, the left SsAC, the left retrosplenial cingulate cortex (RsCC), and the left cingulate cortex (CC) (*p* < 0.03). Data were thresholded at a cluster-extent threshold of *p* ≤ 0.05, both with few corrections and for FDR correction (Figure 1). The results are summarized in Table 3.

#### 3.2.2. Functional Connectivity of the Left Superior Frontal Gyrus

**Figure 2:**
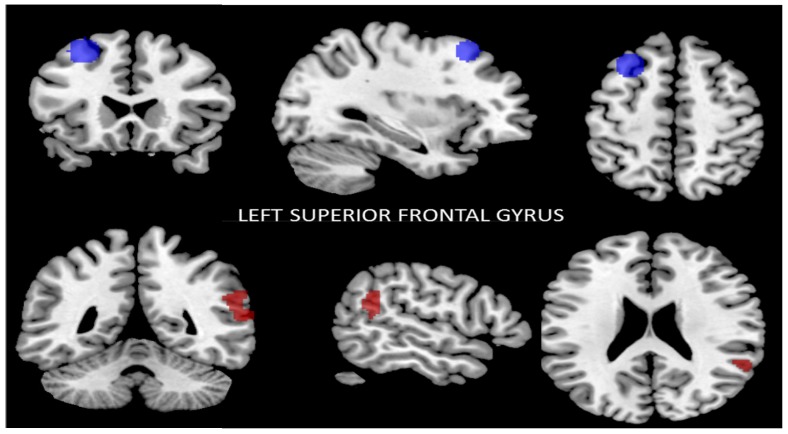
Reduced connectivity between the left superior frontal gyrus (SFG) (blue) and the right supramarginal gyrus (SmG), the right superior temporal gyrus (STG), and the right insular cortex (IC) and right angular gyrus (AG) (red) (*p* < 0.01, FWE-corrected) after the Mediterranean diet and physical activity program compared to baseline. Data are thresholded at a voxel-wise threshold of *p* ≤ 0.05, and a cluster-extent threshold of *p* ≤ 0.05 (FWE-corrected).

The seed-to-voxel analysis showed that after the intervention the left superior frontal gyrus (SFG) region had lower functional connectivity with the right SmG, right STG, right IC, and right AG (*p* ≤ 0.05, FWE-corrected) (Figure 2). The results are summarized in Table 3.

#### 3.2.3. Functional Connectivity of the Right Posterior Superior Temporal Gyrus

**Figure 3:**
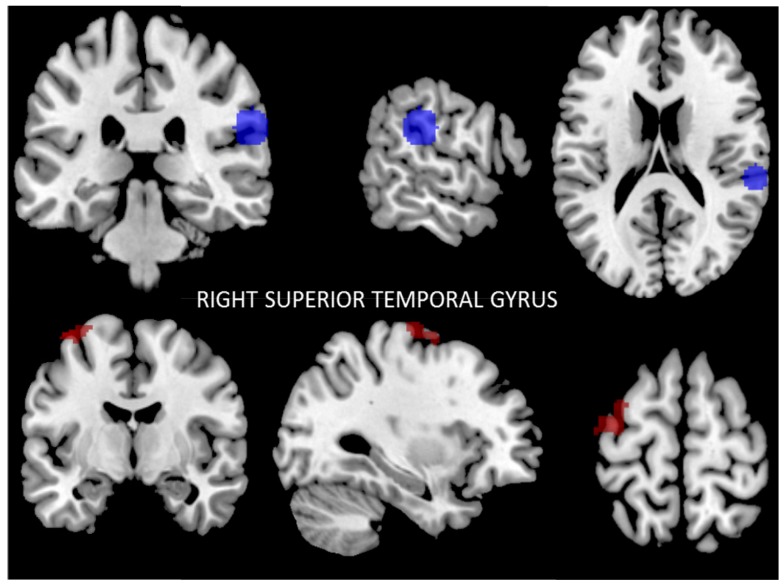
Reduced connectivity between the right superior temporal gyrus (STG) (blue) and the left premotor cortex (PmC), and the left primary motor cortex (PMC) and left primary somatosensory cortex (PSsC) (red) (*p* < 0.02, FWE-corrected) after the Mediterranean diet and physical activity program compared to baseline. Data are thresholded at a voxel-wise threshold of *p* ≤ 0.05, and a cluster-extent threshold of *p* ≤ 0.05, FWE-corrected.

The results of seed-to-voxel analysis showed that after the intervention the right posterior STG region had lower functional connectivity with the left premotor cortex (PmC), left primary motor cortex (PMC), and the left primary somatosensory cortex (PSsC) (*p* < 0.02, FWE-corrected) (Figure 3). The results are summarized in Table 3.

#### 3.2.4. Functional Connectivity of the Right Posterior Cingulate Cortex

In the ROI-to-ROI analysis, the right posterior CC region showed decreased connectivity with the left posterior CC after the program.

**Figure 4:**
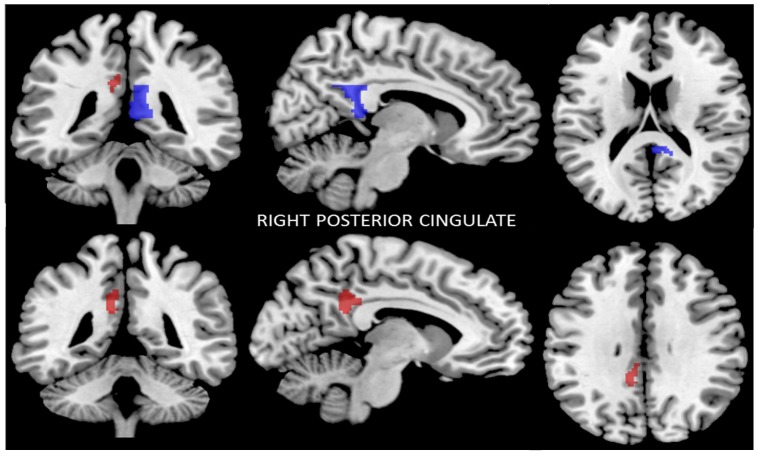
Reduced connectivity between the right posterior cingulate cortex (CC) (blue), the left dorsal posterior cortex (dpCC), and the left ventral posterior cingulate cortex (vpCC) (red) (*p* < 0.04, FWE-corrected) after the Mediterranean diet and physical activity program compared to baseline. Data are thresholded at a voxel-wise threshold of *p* ≤ 0.05, and a cluster-extent threshold of *p* ≤ 0.05, FWE-corrected.

The seed-to-voxel analysis also showed that the right posterior CC had lower functional connectivity with the left dpCC and left ventral posterior cingulate cortex (vpCC) (*p* < 0.04, FWE-corrected) (Figure 4). The results are summarized in Table 3.

We found no statistically significant differences when using the following seed regions: right inferior parietal lobe, right superior frontal cortex, left superior temporal gyrus, left insula, anterior cingulate gyrus, and right and left olfactory regions.

## 4. Discussion

The present study was performed to examine the cerebral functional connectivity in obese women screened before and six months following a program involving a hypocaloric Mediterranean diet and increased physical activity. Previous studies have studied the effect of a Mediterranean diet on cognition and brain structure [7,11]. However, the impact of this intervention program (Mediterranean diet and physical activity) on cerebral connectivity in obese women after weight loss has not been previously studied. After the intervention, the anthropometric variables (BMI and weight) were reduced significantly in all patients, and the blood analysis profile (total cholesterol, LDL cholesterol, insulin resistance index, insulin, and glucose) improved greatly.

The main finding of this study was the identification of decreased connectivity within the inferior parietal cortices, the posterior cingulate, and the prefrontal cortex after the Mediterranean diet and physical activity program (structures included in the DMN). In addition, there was also decreased connectivity within the temporo-insular cortex (included in the SN), as well as decreased connectivity in the somatosensory cortex (included in the SMN) after the intervention program.

Previous studies using functional connectivity analysis of the so-called “default mode network”—a network comprising the lateral parietal cortex, posterior cingulate cortex, and precuneus [33,34]—have demonstrated increased DMN connectivity in overweight/obese individuals, indicating an increased focus on internal states such as appetite or food-related cognitive factors [14,21]. A previous study performed in a eucaloric state found greater connectivity in obese compared to lean individuals in the lateral inferior parietal and posterior cingulate cortices. In addition, the authors found that the lateral parietal connectivity correlated positively with appetite [14]. Thus, greater connectivity between the posterior cingulate cortex has been postulated to contribute to overeating, reflecting an imbalance between cognitive and emotional processing of food cues [21]. In line with previous studies, our results showed decreased connectivity in the left inferior parietal cortex with the posterior cingulate cortices after the intervention program, showing that the connectivity between these structures was modified after the program. As mentioned before, the lateral inferior parietal and posterior cingulates are nodes of the DMN and are involved in coordinating the subsystems of self-referential thought and internal state, such as appetite or other processing of gut signals or food-related cognitive factors [18,35]. Given this fact, it is possible to speculate on the functional significance of over-connectivity of these regions in obese individuals during fasting state [14] and, interestingly, according to our results, this over-connectivity can be rectified by weight loss and physical activity.

On the other hand, we also found decreased connectivity between the lateral inferior parietal cortex and the temporal cortex, including the insula. The insula is part of the so-called “salience network” which is involved in consciousness and self-perception and connects the perception of internal stimuli to aspects of emotion and motivation [36]. The SN, including the anterior cingulate, limbic structures, and temporal cortex, has been related to obesity in that it perceives internal and external cues to appropriately adapt behavior and/or physiology accordingly [37,38]. Thus, the insular cortex plays an established role in cognitive and emotional control and has been suggested to induce the termination of feeding and the ability to predict the satisfactory effects of food intake [39,40]. Increased SN functional connectivity in overweight/obese individuals has also been previously reported [5,15] under resting state conditions. In line with our results, decreased connectivity between the lateral inferior parietal cortex and the temporo-insular cortex after the weight loss program suggest that this decreased connectivity probably plays an inhibiting role in emotional functions susceptible to modification by a weight loss program.

We also found decreased connectivity after the Mediterranean diet and physical intervention program between the prefrontal cortex and the insular/angular gyrus/superior temporal gyrus/supramarginal gyrus. The prefrontal cortex is involved in the central orexigenic network [41], and its connections with the insula reflect an ability to “evaluate” the perception of hunger during fasting state in lean subjects. A “satiation” of this signal is understood to occur after food intake, whereas in obesity there seems to exist a level of insensitivity to these food-related cues [39,42]. According to our results, changes in the connections between the prefrontal and insular cortices are probably implicated in feeding initiative and decrease during weight loss, showing that these regions are also susceptible to modification.

Furthermore, we found decreased connectivity in the temporal cortex with the left premotor, primary motor, and somatosensory cortices after the Mediterranean diet and physical activity intervention program. The SMN—including the supplementary motor area, sensorimotor cortex, and secondary somatosensory cortex [24]—corresponds to the action–execution programs and perception–somesthesis integration [24]. Previous studies have reported evidence for differences in somatosensory processing in obese subjects [25]. Conversely, a recent study in women with anorexia nervosa found decreased connectivity in the SMN when compared with controls, suggesting that it could be related to altered interoception and body image in these women when they are underweight [43]. Our results suggest that changes in sensory-motor structures would be modified after a weight loss program, as these structures play an important role in self-perception and the somesthesis mechanism.

Finally, we found decreased connectivity after the intervention between the right posterior cingulate cortex and the left posterior cingulate cortex, which suggests the integration between both hemispheres [44].

A few studies have examined the effects of different interventions on functional connectivity in individuals with obesity. McFadden et al. found a six-month exercise intervention to be associated with reduced DMN connectivity in the precuneus during rest. Interestingly, the authors concluded that, given previous reports of DMN overconnectivity in overweight/obese individuals, the findings may indicate an exercise-related “normalization” of network function [16]. Recently, Legget et al. found changes in connectivity after a six-month exercise program in the posterior cingulate cortex and other networks (language, visual, sensorimotor, executive, basal ganglia, and posterior DMN), which were significantly reduced [45]. Other authors have found evidence of changes in functional connectivity depending on intervention type (diet vs. bariatric surgery) after weight loss [17].

The effects of weight loss on the brain are still unknown. It is difficult to speculate which biological changes are associated with the differences observed during our study in functional brain connectivity after the Mediterranean diet and physical activity program. Exercise has been suggested to promote neuroprotection, neurogenesis, and neuroplasticity [16,46,47,48]. Exercise may affect metabolic factors and neuronal circuitry and exert positive effects on neuronal networks by reducing the expression of inflammatory cytokines, increasing the production of neurotrophic factors, or increasing neurotransmitter levels [16,46,47,48]. In addition, weight loss also influences adipocytokines, inducing lower levels of leptin and higher levels of adiponectin. Adiponectin—levels of which are inversely correlated with adiposity—improves insulin sensitivity in major insulin target tissues, modulates inflammatory responses, and plays a crucial role in the regulation of energy metabolism. Previous studies have suggested neuroprotective effects of adiponectin in the hippocampal cells in the temporal lobe [49,50]. The burden of the insula and the precuneus has also been associated with low levels of adiponectin [51], and both these areas are important structures of the main brain networks (DMN and SN).

The present study is the first to study resting-state functional connectivity in obesity while including both a Mediterranean diet and physical activity intervention. Several limitations of the current study need to be addressed. First, the sample size is small, which is a potential limitation. Another important limitation is that there was no control group to safely attribute changes, though the results and the longitudinal design are strengths. In addition, it is not possible to know whether the changes are due to physical exercise, the Mediterranean diet reducing caloric intake, or both. In general, patients carrying out a weight loss program at the Department of Endocrinology are advised to follow a diet (reducing caloric intake) as well as engaging in physical exercise. Further longitudinal studies including only physical exercise [16] would be desirable to determine which is responsible for the connectivity changes—the Mediterranean diet, the physical activity, or both. Moreover, only female subjects were included. We decided to include only female subjects to avoid a sex bias. As a consequence, our results can only be generalized to women. Finally, other technical limitations (e.g., using a six-channel head coil rather than the more standard 12-channel head coil which would have reduced the noise ratio) could influence the findings. Furthermore, all the functional MRIs (pre- and post-intervention) were carried out under fasting conditions, and it would be interesting to evaluate brain connectivity in a state of satiety; it is probable that the brain networks found would differ since previous evidence shows that the pathophysiology is different [52,53]. Despite the aforementioned limitations, we still consider our results to be clinically pertinent, as they support the benefits of the Mediterranean diet and may provide a better understanding of the pathophysiology of obesity. However, more longitudinal studies on larger samples, with an appropriate design including a control group, are needed to shed light on the effects of obesity on the brain and to aid in developing future treatments that could act on the neural networks involved in this disease.

In conclusion, our findings suggest that functional connectivity between specific brain structures involved in obesity physiopathology (the inferior parietal lobe, posterior cingulate, temporo-insular cortex, and prefrontal cortex) might be modified by a weight loss program including a Mediterranean diet and physical exercise.

## Figures and Tables

**Table 1:** Clinical, anthropometric, and blood analysis characteristics (mean ± standard deviation).

	Before the Mediterranean Diet/Physical Activity Program	After the Mediterranean Diet/Physical Activity Program
*N* (women)	16	
Age (years)	46.3 ± 4.07	
Weight (kg)	98.5 ± 13.1	88.2 ±12.2 *
Body mass index (kg/m^2^)	38.1 ± 4.7	34.1 ± 4.5 *
Waist circumference(cm)	115.3 ± 11.8	110.5 ± 10.9
Glucose (mg/dL)	92.9 ± 12.5	82.5 ± 12.9 *
HbA1c (%)	5.5 ± 0.4	5.4 ± 0.3
Creatinine (mg/dL)	0.6 ± 0.1	0.6 ± 0.1
Uric acid (mg/dL)	13.6 ± 34.0	5.0 ± 0.7
Total cholesterol (mg/dL)	202.1 ± 38.2	182.8 ± 34.7 *
LDL cholesterol (mg/dL)	124.1 ± 33.4	109.4 ± 33.6 *
HDL cholesterol (mg/dL)	56.0 ± 9.7	50.8 ± 10.7
Triglycerides (mg/dL)	93.50 (73.75–122.50)	94.50 (74.00–150.5)
Insulin (µIU/mL)	15.4 ± 5.5	11.5 ± 5.1 *
HOMA-IR index	3.5 ± 1.3	2.3 ± 0.9 *

* *p* ≤ 0.05 for comparison before and after the intensive weight loss program. HbA1c, glycated hemoglobin; HDL, high-density lipoprotein; HOMA-IR, insulin resistance index; LDL, low-density lipoprotein.

**Table 2:** Changes in energy and nutrient intake according to weight loss, baseline, and six months after intervention.

	Weight Loss	Baseline	6 Months	*p*
Energy (kcal)	<5%	2537.9 ± 100.3	1909.1 ± 70.9	<0.0001
	≥5–<10%	2440.8 ± 157.1	1658.2 ± 90.8	0.001
	≥10%	2339.8 ± 79.0	1621.0 ± 48.6	<0.0001
Energy from total carbohydrate (%)	<5%	250.1 ± 12.8	181.2 ± 7.4	<0.0001
	≥5–<10%	246.1 ± 16.1	169.7 ± 14.6	0.005
	≥10%	230.3 ± 10.5	172.9 ± 5.8	<0.0001
Energy from total protein (%)	<5%	95.9 ± 3.6	83.6 ± 3.6	0.01
	≥5–<10%	92.3 ± 3.9	76.4 ± 3.3	0.001
	≥10%	89.4 ± 3.1	72.6 ± 2.1	0.001
Energy from total fat (%)	<5%	122.0 ± 5.2	92.9 ± 4.5	<0.0001
	≥5–<10%	124.6 ± 7.7	72.6 ± 4.4	0.001
	≥10%	115.6 ± 4.9	70.4 ± 4.1	<0.0001
SFA	<5%	36.7 ± 2.3	25.6 ± 1.7	<0.0001
	≥5–<10%	38.2 ± 3.1	18.9 ± 1.3	<0.0001
	≥10%	44.6 ± 10.1	17.9 ± 1.5	<0.0001
MUFA	<5%	59.3 ± 2.3	62.0 ± 14.1	<0.0001
	≥5–<10%	59.4 ± 3.3	38.2 ± 2.6	0.001
	≥10%	55.4 ± 2.4	37.4 ± 2.2	<0.0001
PUFA	<5%	17.2 ± 1.5	11.3 ± 0.6	<0.0001
	≥5–<10%	18.6 ± 2.1	9.7 ± 0.9	<0.0001
	≥10%	16.5 ± 1.3	10.1 ± 0.8	0.001
Cholesterol (mg/d)	<5%	382.5 ± 24.7	334.0 ± 25.6	0.3
	≥5–<10%	417.6 ± 26.5	238.9 ± 22.6	0.001
	≥10%	395.7 ± 22.2	232.1 ± 12.9	<0.0001
Fiber (g/d)	<5%	21.9 ± 1.5	18.8 ± 1.2	0.3
	≥5–<10%	18.9 ± 1.2	20.2 ± 1.3	0.6
	≥10%	17.2 ± 1.0	23.5 ± 1.4	0.001
Vitamin D (µg/d)	<5%	3.7 ± 0.5	6.3 ± 1.2	0.3
	≥5–<10%	2.3 ± 0.4	6.1 ± 1.2	0.01
	≥10%	3.7 ± 1.3	9.2 ± 3.3	0.004

SFA, saturated fatty acid; MUFA, monounsaturated fatty acid; PUFA, polyunsaturated fatty acid.

**Table 3:** Seed-to-voxel results: left inferior parietal lobe, left superior frontal gyrus, right posterior superior temporal gyrus, and right posterior cingulate cortex connectivity.

**Seed**	**Left Inferior Parietal Lobe Functional Connectivity** **MNI (x, y, z): (−42, −68, 38)**			
Cluster 1	Brodmann Area	MNI (x, y, z)	Cluster Size (K)	*p*-value (FWE)
R AG	39	+50, −46, +28	136	<0.001
R STG	22		110	
R SmG	40		85	
R IC	13		25	
Cluster 2	Brodmann area	MNI (x, y, z)	Cluster size (K)	*p*-value (FWE)
R DPCC	31	+16, −56, +30	170	<0.001
R SsAC	7		169	
L SsAC	7		100	
Cluster 3	Brodmann area	MNI (x, y, z)	Cluster size (K)	*p*-value (FWE)
L DPCC	31	–08, −60, +36	63	<0.06 <0.03 (FDR)
L SsAC	7		30	
L RsCC	29		15	
L CC	30		5	
**Seed**	**Left Superior Frontal Gyrus Functional Connectivity** **MNI: (−28, 22, 52)**			
	Brodmann area	MNI (x, y, z)	Cluster size (K)	*p*-value (FWE)
R SmG	40	+64, −50, +12	68	<0.01
R STG	22		61	
R IC	13		18	
R AG	39		14	
**Seed**	**Right Posterior Superior Temporal Gyrus (pSTG) Functional Connectivity** **MNI (x, y, z): (60, −30, 24)**			
	Brodmann area	MNI (x, y, z)	Cluster size (K)	*p*-value (FWE)
L PmC	6	−36, −10, +68	137	<0.025
L PMC	4		29	
L PSsC	3		9	
**Seed**	**Right Posterior Cingulate Cortex Functional Connectivity** **MNI (x, y, z): (0, 6, 40)**			
	Brodmann area	MNI (x, y, z)	Cluster size (K)	*p*-value (FWE)
L dpCC	31	−10, −46, +24	120	<0.04
L vpCC	23		2	

Abbreviations: L, left; R, right; AG, angular gyrus; SmG, supramarginal gyrus; IC, insular cortex; DpCC, dorsal posterior cingulate cortex; SsAC, somatosensory association cortex; RsCC, retrosplenial cingulate cortex; CC, cingulate cortex; SFG, superior frontal gyrus pSTG, posterior superior temporal gyrus; PmC, premotor cortex; PMC, primary motor cortex; PSsC, primary somatosensory cortex; pCC, posterior cingulate cortex; vpCC, ventral posterior cingulate cortex; MNI Montreal Neurological Institute; FWE, family wise error; FDR, false discovery rate.
